# Supplementary material for: Easier said than done: unexpected hurdles to preparing ∼1,000 cranial CT scans for data collection from an online digital repository
Source: PeerJ. 2025 Oct 20;13:e20172. doi: 10.7717/peerj.20172 (PMC12548635; doi:10.7717/peerj.20172)
Supplement: Supplementary file 1 [file peerj-13-20172-s001.odt]

## Supplemental Information

Supplemental Information Figure 1. Normal loading of a DICOM file.

Supplemental Information Figure 2. Multivolume creation when skull W584 was loaded.

Supplemental Information Figure 3. Example of a volume comprised of 6 slices.

Supplemental Information Figure 4. Parameters to load the volumes with warnings.

Supplemental Information Figure 5. Warning message

Supplemental Information Figure 6. Problematic skull with 0.58 mm spacing

Supplemental Information Figure 7. Segmented models of the baboon skull W584 loaded in 3D Slicer. a) 0.584 mm slice spacing. b) 0.60 mm slice spacing. c) comparison of orthoslices between 0.58 mm slice spacing (in green) and 0.60 mm slice spacing (in black and white); left: frontal plane; center: transverse plane; right: sagittal plane. Note that changing the slice spacing greatly affects the length of the skull, but the deformation is much less evident in the width and the depth of the skull.
